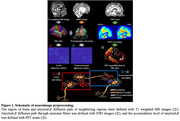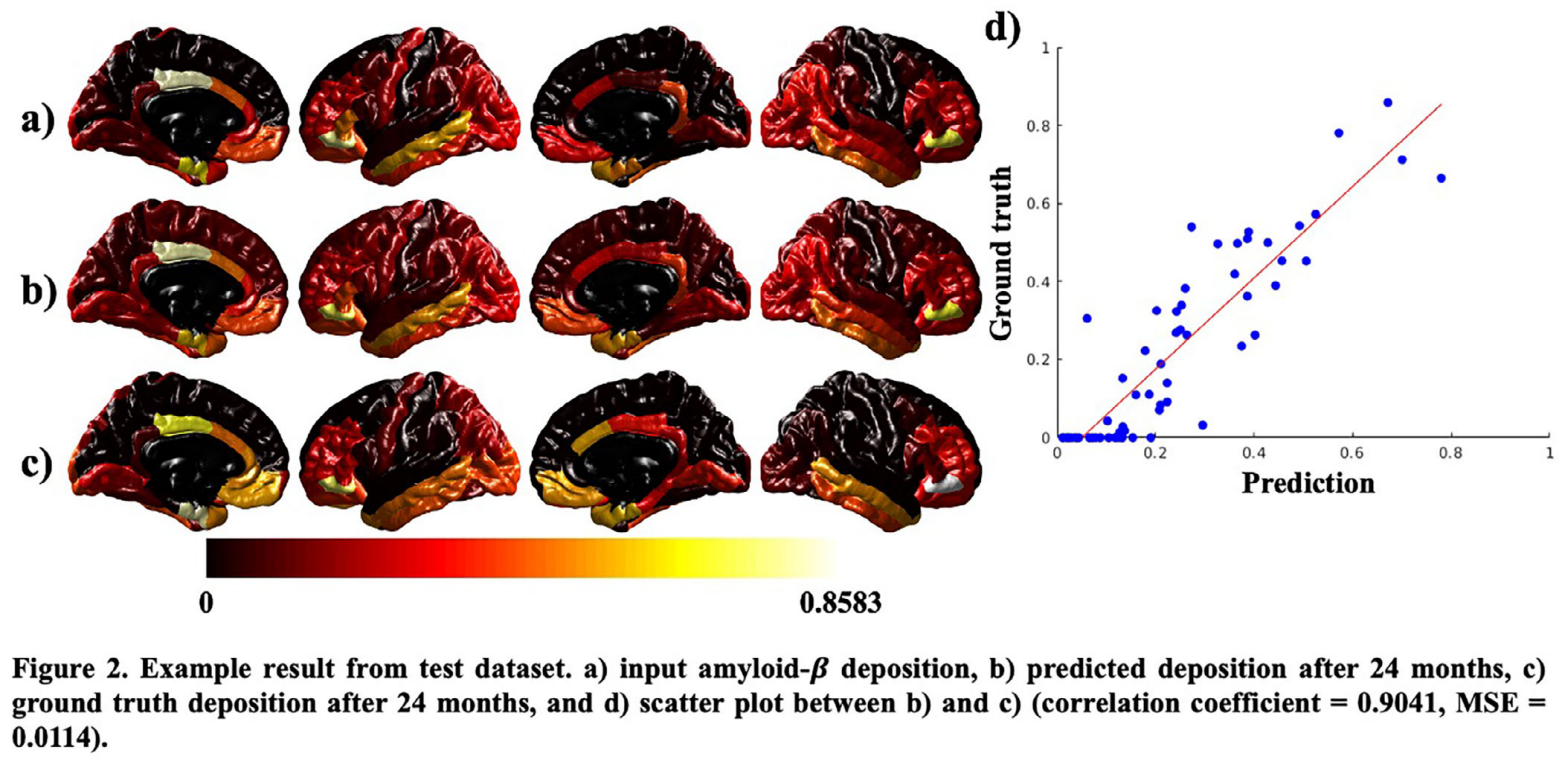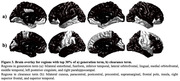# Deep Learning Model for Forecasting Propagation of Amyloidopathy in Alzheimer’s Disease

**DOI:** 10.1002/alz.085828

**Published:** 2025-01-03

**Authors:** ByeongChang Jeong, Daegyeom Kim, Surin Seo, Seung‐Hoon Lee, Hyun‐Ghang Jeong, Cheol E Han, The Alzheimer’s Disease Neuroimaging Initiative

**Affiliations:** ^1^ Korea University, Sejong, Sejong Korea, Republic of (South); ^2^ Korea University College of Medicine, Seoul, Seoul Korea, Republic of (South); ^3^ Korea University Guro Hospital, Korea University College of Medicine, Seoul, Seoul Korea, Republic of (South)

## Abstract

**Background:**

Amyloid‐β accumulation is a pivotal factor in Alzheimer’s disease (AD) progression. As treatment for AD has not been successful yet, the most effective approach lies in early diagnosis and the subsequent delay of disease progression. Hence, this study introduces a deep learning model to predict amyloid‐β accumulation in the brain.

**Method:**

We mathematically modeled the diffusion of amyloid‐β based on its biological traits, encompassing generation, clearance, and diffusion. We converted the model into a deep learning framework with multi‐layer perceptron (MLP) and graph convolutional neural network (GCN) (Kipf et al., 2016) to forecast the accumulation of the protein. We extracted the necessary information from various neuroimage data, including T1 structural magnetic resonance (MR) images, ^18^F‐Florbetapir positron emission tomography (PET) scans, and diffusion weighted MR images (DWI), to simulate the diffusion of the protein (Figure 1). We used longitudinal data of 146 subjects, incorporating 436 data points.

**Result:**

The proposed model accurately predicted amyloid‐β after 2 years (Figure 2), showing a high correlation in the test dataset (median = 0.8273, IQR = [0.7708, 0.8692]), outperforming the previous model (average 0.58) (Kim et al., 2019). We examined generation and clearance terms, mapping top 30% ROIs onto the brain by averaging each term across subjects (Figure 3). The regions with early AD amyloid‐β accumulation are believed to be related to the default mode network and prefrontal network (Palmqvist et al., 2017) supported by Figure 3a. The effectiveness of amyloid‐β clearance may be influenced by brain activity (Mergenthaler et al., 2013; Ullah et al., 2023). Earlier studies reported diminished metabolism in specific regions during the early AD (Chételat et al., 2020; Kantarci et al., 2021). The proposed model identified high clearance regions (Figure 3b), aligning with regions showing normal metabolism.

**Conclusion:**

We introduced a deep learning model that simulates the diffusion of amyloid‐β with strong predictive performance and interpretation. While parameters were optimized for the entire group, accuracy varied for some subjects. Also, further investigation is needed to interpret each term comprehensively. Despite the need for individual optimization and additional interpretative analysis, the model may contribute to the diagnosis of AD.